# Northern Peatlands in Transition in the 21st Century – Land Use, Status, Policies and Future Trajectories: Comparisons Between Finland, Ireland and Scotland

**DOI:** 10.1007/s00267-025-02376-y

**Published:** 2026-01-30

**Authors:** Maija Lampela, Florence Renou-Wilson, Roxane Andersen, David Wilson, Rebekka R. E. Artz, Hannah Clilverd, Jukka Turunen, Anne Tolvanen, Liisa Maanavilja, Anna M. Laine

**Affiliations:** 1 Environmental Solutions, Geological Survey of Finland, Espoo, Finland; 2 School of Biology and Environmental Science, University College Dublin, Dublin, Ireland; 3 Environmental Research Institute, University of the Highlands and Islands, Thurso, UK; 4 Earthy Matters Environmental Consultants Co., Donegal, Ireland; 5 Ecological Sciences, The James Hutton Institute, Aberdeen, UK; 6 UK Centre for Ecology and Hydrology, Penicuik, UK; 7 Natural Resources Institute Finland (Luke), Oulu, Finland; 8 Environmental Solutions, Geological Survey of Finland, Kuopio, Finland; 9 School of Forest Sciences, University of Eastern Finland, Joensuu, Finland

**Keywords:** Peatlands, Mires, Peat, National Peatland Strategies, Restoration, Rewetting

## Abstract

Climate change and biodiversity losses have necessitated innovative approaches to peatland management. This study examines pivotal historical landmarks and the recent forces of change that have affected peatlands in Finland, Ireland and Scotland, highlighting how national contexts, such as land ownership, forestry, agriculture and the need for domestic energy sources, have shaped the peatland use in those countries. We further introduce national and EU policies, which include, for example, national peatland strategies, and identify barriers to sustainable management of these important ecosystems. We propose six key solutions that could improve peatland persistence more broadly in northern Europe: (1) adoption of an integrated, landscape-scale strategy for rewetting and restoration with multi-stakeholder collaboration, (2) enhancement of monitoring to improve outcomes and refine best practices, (3) alignment of both national and EU policies across relevant sectors (energy, climate change, biodiversity, land use) to promote sustainable peatland management, (4) minimisation of trade-offs between green energy transition and sustainable peatland management, (5) engagement with local communities in restoration efforts for better acceptability and outcomes, and (6) wider leverage of market-based mechanisms, such as carbon, biodiversity and water credits, to finance peatland restoration. Together, these measures provide a pathway for the sustainable management of northern peatlands by balancing environmental integrity with socio-economic needs.

## Introduction

Despite their relatively small global coverage (3%), peatlands store an estimated ~600 Gt of carbon (Yu et al. 2010), which makes them disproportionately significant in global climate regulation (Frolking et al. 2011, Joosten et al. 2016). However, for centuries, peatlands in most countries have been subject to a wide range of land use pressures, such as drainage for agriculture and forestry. This has led to an ongoing decline in the ecosystem services that peatlands provide, such as climate regulation, provision of biodiversity/habitats (Andersen et al. 2013, Grzybowski & Glińska-Lewczuk 2020, Räsänen et al. 2023), and the maintenance of water quality and resources in peatland-dominated catchments (Tiemeyer et al. 2007, Härkönen et al. 2023, Pschenyckyj et al. 2023).

**Fig. 1:**
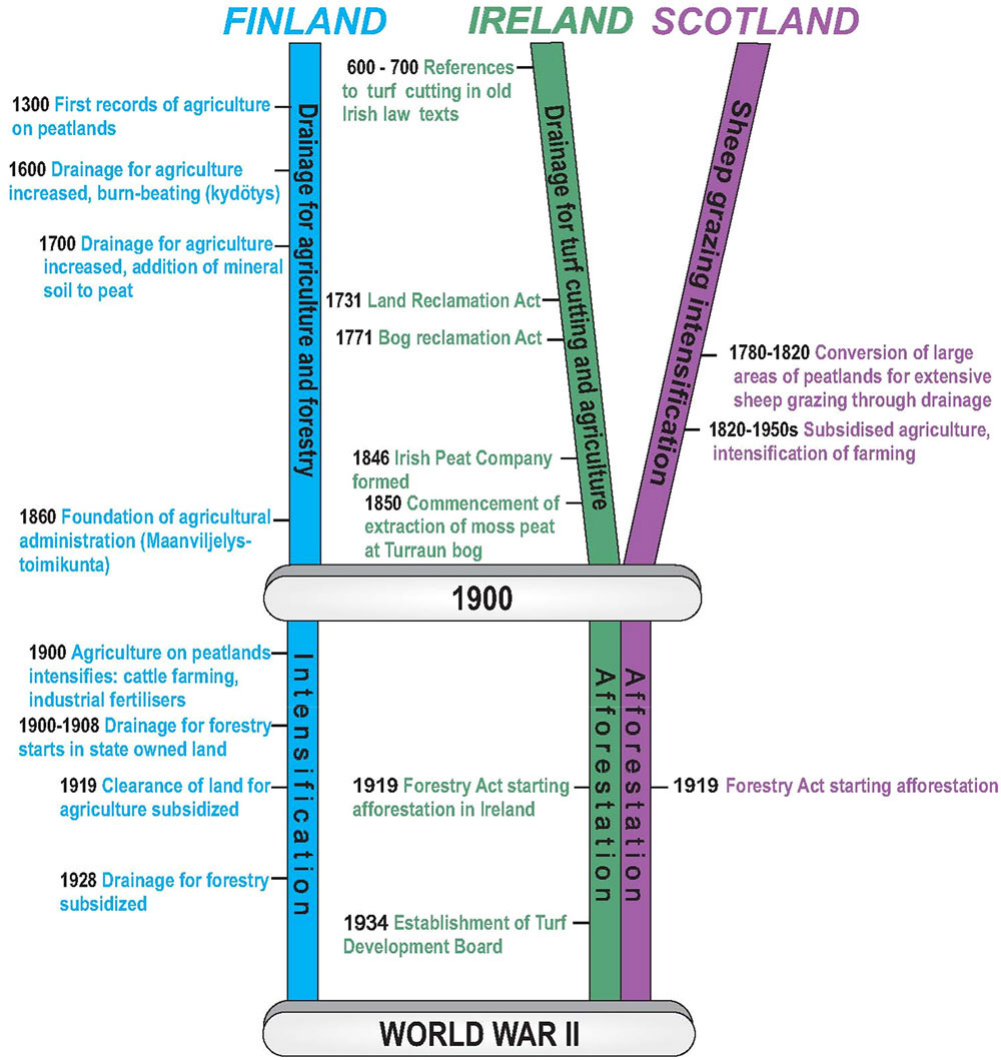
Timeline of peatland land use in Finland, Ireland and Scotland from early exploitation through to World War II

The regional importance of peatlands is extremely variable, and in some northern European countries, such as Finland, Ireland and Scotland^1^, peatlands can cover up to 30% of the land surface, making them a key component of the national landscape. Over the centuries, the use of peatlands in these countries has been intensive, with substantial portions drained for agriculture, forestry and peat extraction (Table 1 and Fig. 1). Peatland drainage leads to large and persistent soil greenhouse gas (GHG) emissions (Wilson et al. 2016) and increases the export of dissolved organic carbon (DOC) and particulate organic carbon (POC) from peatlands to adjacent water bodies (Carlson et al. 2017, Jauhiainen et al. 2019). The extent of drainage impacts on GHG emissions and biodiversity depends on the land-use, with peat extraction and crop production showing greater negative impacts than forestry drainage, particularly in peatlands that were already forested at the time of drainage (Regina et al. 2019, Jauhiainen et al. 2023).

**Table 1:** Area of organic soils in 2021 disaggregated by land use (in kha = 1000 ha), according to United Nations Framework Convention on Climate Change (UNFCCC 2021) reporting requirements

	Finland	Ireland	Scotland
	Area (kha)	Area (kha)	Area (kha)
Cropland, annual plants (cereals)	106	1	5
Cropland, perennial plants (grass)	173	-	^a^
Grassland	63^b^	339	103^c^
Grassland (Scotland only): modified bog, not sown.			892
Forest land	5963^d^	459	360
- of which drained	4313^e^	459	360
Peat extraction (industrial)	101	41	2
Peat extraction (domestic)		84	45
Rewetted peatland	55	85	48
Reservoirs and human-made impoundments	13	-	-
Settlements	17	3	6
**Total reported peatland area under different land uses**	**6491**	**1012**	**1461**
Near-natural	2609	448	491
**Estimated current peatland area**	**9100**	**1460**	**1952**

Data for Scotland are derived from Brown et al. (2023). Data for Ireland are derived from EPA (2024). Estimated current peatland areas are taken from Turunen & Valpola (2020) for Finland, Tanneberger et al. (2017) for Ireland, and Brown et al. (2023) for Scotland

^a^Included under Cropland

^b^In Finland, grassland mainly consists of abandoned fields

^c^This value has very high uncertainty. A recent field validation suggests that a 2–20 kha area is more likely, with the remainder most likely to be modified bog (i.e., land cover that has more heath-like characteristics rather than grassland or near-natural mire vegetation). This could be due to land use deintensification over recent decades and/or classification difficulties in broader land cover schemes

^d^In Finland, some forested peatlands are in use without drainage

^e^In Finland, the original area of peatlands drained for forestry (c. 5.7 Mha) has decreased due to the full loss of peat in originally shallow peat areas

**Fig. 2:**
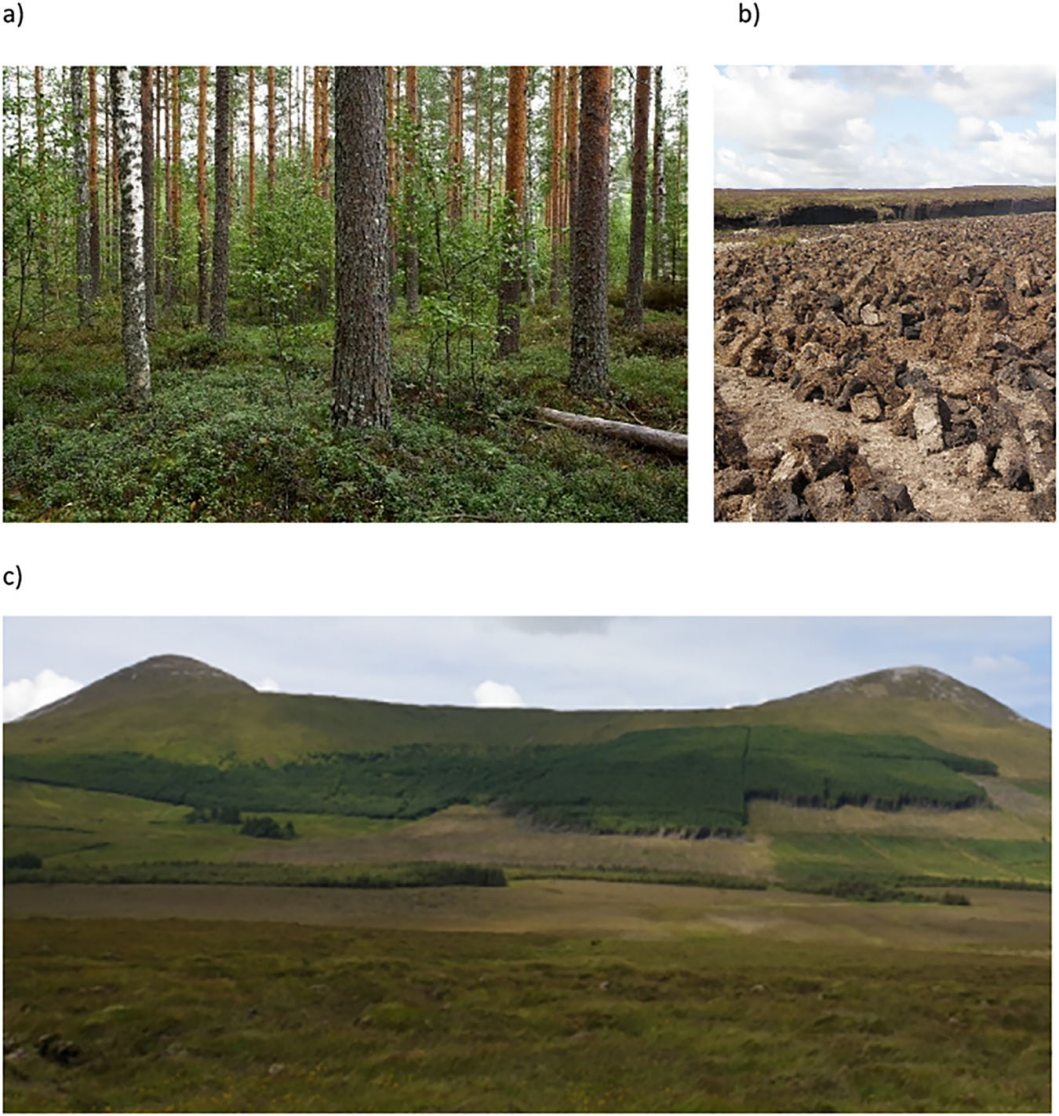
**a** Forestry-drained peatland in southern Finland (photo: Jukka Laine), **b** peat cutting in Orkney, Scotland (photo: Steven White), **c** afforested blanket bog in Ireland (photo: Florence Renou-Wilson)

Although drainage and reclamation of peatlands have been viewed as emblematic of technical and socio-economic progress in Finland, Ireland and Scotland, each country has followed a different path in peatland management, land use practices and outcomes, as reflected in their current land use status (Table 1). In Finland, the focus has been on forestry drainage, while in Ireland, domestic turf cutting, industrial peat extraction and plantation forestry have dominated (Fig. 2). In Scotland, grazing, plantation forestry, and more recently, wind farms have been the primary land uses (Renou-Wilson 2018, Brown 2020, Heal et al. 2020, Turunen & Valpola 2020).

Recent European energy and environmental policies aimed at addressing climate change and biodiversity loss have shifted public perception of peatlands and the potential for their sustainable management^2^ (Flood et al. 2022). While there is increasing public support for better outcomes, stakeholder consensus on the means to achieve these outcomes remains elusive (Tolvanen et al. 2013, O’Riordan et al. 2016, Byg et al. 2017). In this paper, we examined peatland land-use histories, policies and future prospects in Finland, Ireland and Scotland. The significance of peatlands in these three countries led to an early recognition of the need for sustainable management, materialised in the creation of the first national peatland strategies globally. Here, we evaluated the ecological, political, and socio-economic barriers to sustainable peatland management, concentrating on protection and rewetting/restoration, and recommend solutions, based on best practices across the countries, to enhance the sustainable management of these important ecosystems.

## Historical Overview of Peatland Use

**Fig. 3:**
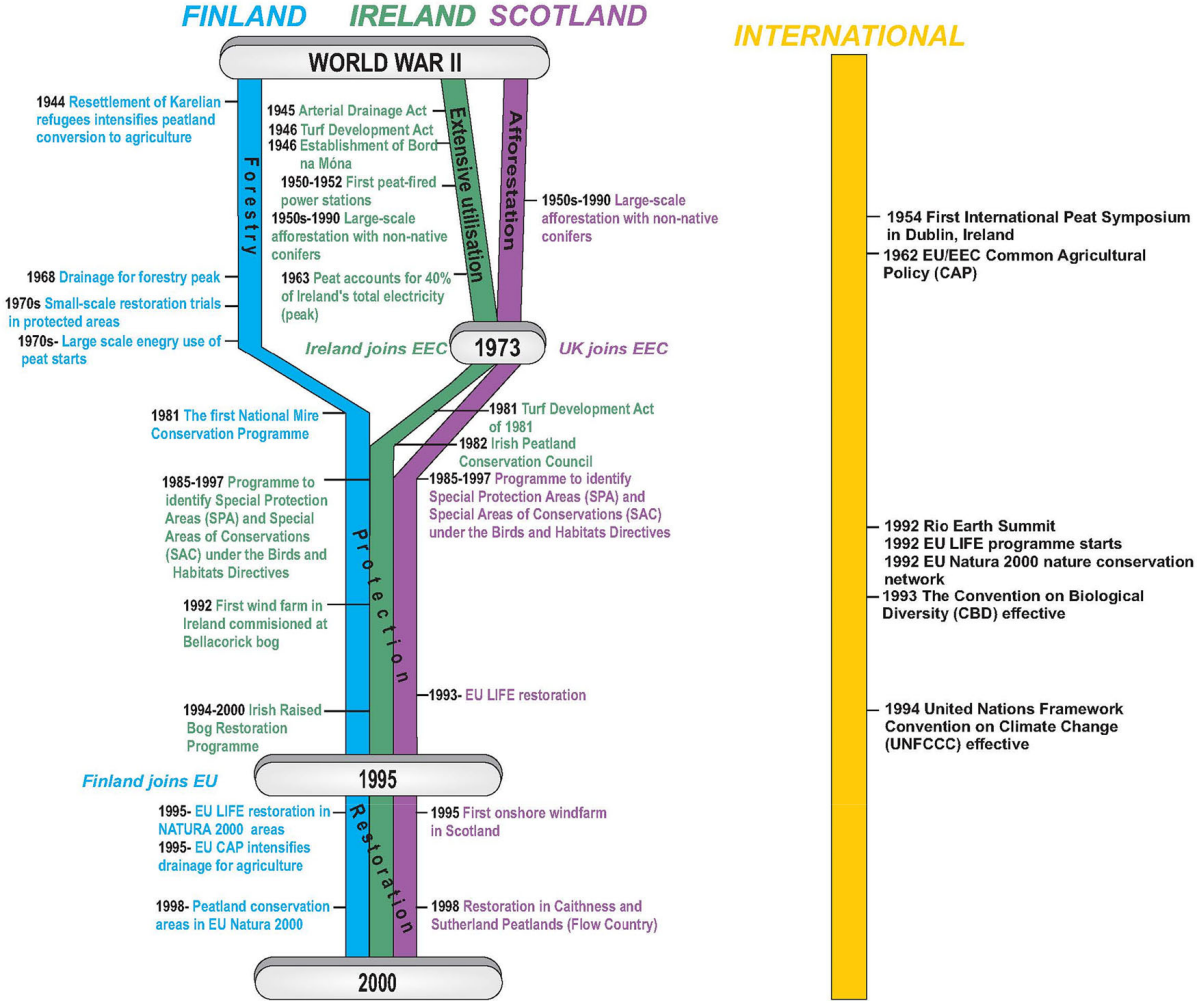
Timeline of peatland land use in Finland, Ireland and Scotland from World War II to 2000

**Fig. 4:**
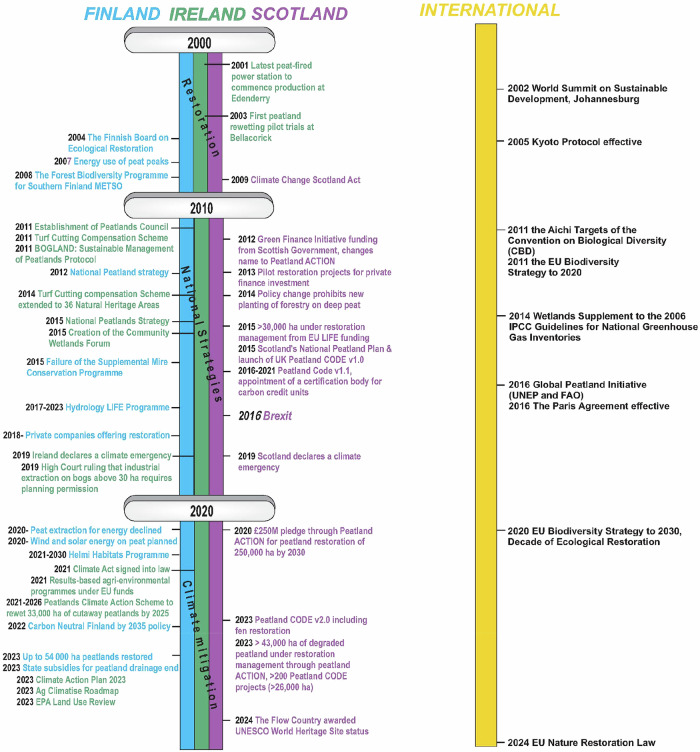
Timeline of peatland land use in Finland, Ireland and Scotland from 2000 to the present

Historically, peatlands have been viewed as unproductive wastelands, sometimes even as dangerous or mythical places, which require “improvement” to become productive and civilised. Traditional land uses such as agriculture, grazing and turf cutting for domestic use on peatlands in Finland, Ireland and Scotland have had ecosystem-altering and degrading effects (Fig. 1). Since the 18th century, population growth and industrialisation have affected peatland land-use, increasing the levels of reclamation and degradation. During the 20th century (and especially after WWII), further land use changes were associated with peatland forestry, afforestation and industrial peat extraction for energy and horticultural use. European Economic Community (EEC) and European Union (EU, succeeding EEC) membership in 1973 for UK and Ireland and 1995 for Finland incentivised agriculture on peatlands, and a rising awareness of environmental issues strongly influenced peatland conservation (Fig. 3). In the 21st century, the impacts of climate change, increasing peatland degradation, as well as rising incentives for restoration and sustainable land-use have further shaped peatland management (Fig. 4). This long history of utilisation, combined with policy steering towards intensive use and scattered (as in Finland and Ireland) or highly concentrated (Scotland) landownership, have created barriers to sustainable peatland use.

### Finland

Excluding the Russian Federation, the largest area of peatlands in Europe is found in Finland (Tanneberger et al. 2017). In the 1950s, the total peatland area of the country was estimated at 10.2 million ha (Mha). Since then, approximately 1 Mha of peatlands, originally with shallow peat layers (<30 cm peat depth, ≥30% organic matter), have been transformed (through oxidation of the drained peat) into mineral soils (Table 1). Currently, the total peatland area of the country is estimated at 9.1 Mha, comprising 27% of the land area. These peatlands store an estimated 5079 Mt of carbon (Turunen and Valpola 2020). To date, approximately 5.7 Mha of peatlands have been drained for forestry, 0.3 Mha for agriculture and 0.1 Mha for peat extraction (Turunen and Valpola 2020) and approximately 55,000 ha have been rewetted or restored (Metsähallitus 2023, personal communication).

The first records of Finnish peatland utilisation are from the 14th century (Fig. 1). Peatland drainage for agriculture increased gradually with new technologies, such as the burn-beating (burning the topsoil to reduce acidity) in the 17th century and the addition of mineral soils to the peat in the 18th century (Kunnas 2005). Agricultural use expanded at the turn of the 20th century, driven by the growing need for cattle farming and fodder production (Pykälä 2001), and from 1919 onwards, the clearance of peatlands for cultivation was subsidised by the Finnish State. During the 20th century, peatland conversion to agriculture was driven both by population growth and by the resettlement programme for Karelian refugees after WWII (Fig. 3). Another wave of peatland drainage for agriculture started after 1995, when Finland joined the EU, and the Common Agricultural Policy (CAP) promoted dairy production in peat-rich regions (Regina et al. 2016).

Forestry drainage (Fig. 2a) in Finland started in the early 20th century to support the growing forest industry. Drainage peaked in the 1960s and 1970s, resulting in more than half of the country’s peatland area being drained for forestry by the 1980s (Päivänen 2007). Since 1928, forestry drainage on private land has been subsidised by the State through the Sustainable Forestry Financing Act. From 1997 onwards, funding has been provided only for maintenance drainage of previously drained land (Ollonqvist 2004). These subsidies were discontinued at the end of 2023.

The global oil crisis in 1973 stimulated large-scale peat extraction, led by the state-owned company Vapo. Since then, peat energy use has been supported by numerous national policies that have included tax reductions and feed-in tariffs, partly counteracting the effects of the EU Emissions Trading System introduced in 2005, which was aimed at reducing GHG emissions from fuels. Annual peat energy consumption peaked in 2007, accounting for 7% of the country’s total energy use, but has since declined to 1.5% in 2024 (Statistics Finland 2025).

### Ireland

In the Republic of Ireland (hereafter Ireland), peatlands cover 1.46 Mha, or 21% of the land area, the second-highest proportion in Europe after Finland (Tanneberger et al. 2017). However, more recent mapping work would suggest that if a broader definition were used (≥10 cm peat depth and ≥8.6% organic matter), peat soils would cover an area of 1.66 Mha (an increase of 13%) (Gilet et al. 2024). Extensive land use change has taken place in peatlands over the last centuries, but particularly over the last three decades (1990–2020) (Habib and Connolly 2023). The land use distribution reported to the UNFCCC is as follows (Table 1): 0.46 Mha under plantation forestry, 0.34 Mha under grasslands, 0.08 Mha for domestic turf cutting, and 0.08 Mha for industrial peat extraction (Aitova et al. 2023). The area of undrained or ‘near-natural’ peatlands is estimated at 0.45 Mha. To date, 85,000 ha have been officially rewetted and mostly include rehabilitated industrial cutaway peatlands. Irish peatlands are estimated to store c. 2216 Mt of carbon with near-natural raised bogs containing 3037 t carbon/ha (Renou-Wilson et al. 2022).

Irish peatlands have been utilised since early medieval times, with considerable land use pressure mounting over the centuries, mainly driven by publicly funded schemes. From 1716 onwards, a series of Governmental Acts (Fig. 1) were passed to promote peatland reclamation. As deforestation reached its peak in the late 18th century, peat became the only available indigenous fuel (Feehan et al. 2008). By the 1840s, with the population rising to 8.2 million, peatlands were also drained for grazing and agriculture. After independence in 1922, Ireland established the Turf Development Board in 1934, which became Bord na Móna in 1946. In parallel, peatland drainage for agriculture increased through several Acts, such as the 1945 Arterial Drainage Act, and the 1981 Programme for Western Development, which represented the largest land use change over time (Feehan et al. 2008). In the 1980s and 1990s, the EU Headage grant scheme intensified sheep grazing on blanket bogs. At the same time, considerable areas of blanket bogs were afforested (Fig. 2c) by the Irish state forestry board, Coillte (Renou-Wilson and Byrne 2015, Renou and Farrell 2005). Although afforestation of blanket bogs has officially declined, private afforestation on former farmed peat soils continues.

The 20th century saw a plethora of technological developments to industrially extract peat for electricity production, while stimulating private turf (i.e., peat that had been cut by hand and dried for fuel) production. A grant aid scheme under the Turf Development Act 1981, together with the increasing demand for horticulture peat, enabled many small-scale extraction programmes to be carried out on thus far undrained smaller bogs. Peak electricity generation from peat to the Irish grid occurred at the end of the 20th century due to State subsidies, which ended in 2020. The last peat-burning power station had switched to 100% biomass use by the end of 2023. However, domestic peat extraction remains a consistent land use pressure (recent work by Aitova et al. 2023 estimated that approximately 22% of Irish peatlands have been impacted by domestic extraction), particularly on blanket bogs and, despite EU fines, on protected Special Areas of Conservation (SAC) and National Heritage Areas (NHA). A remote sensing analysis revealed that 30% of Irish peatlands have undergone major land use change between 1990 and 2019, mainly due to increased forest cover and a decline in industrial bare cutaway peatlands (Habib and Connolly 2023).

### Scotland

The extent of peatlands in Scotland is currently estimated at 1.95 Mha (≥50 cm peat depth and >35% organic matter, following Minasny et al. 2024), which represents 25% of the land area of the country (Brown et al. 2023). Scottish peat soils contain an estimated 1889 Mt carbon (Aitkenhead and Coull 2020). It is estimated that up to 80% of Scottish peatlands have been altered by centuries of human activity, including turf cutting for fuel, burning, grazing, drainage, afforestation and, more recently, windfarm development. The currently estimated distribution of peatlands by land use category is as follows: 0.9 Mha under modified bogs (affected by overgrazing, drainage or burning), 0.36 Mha under plantation forestry (also very uncertain due to peat depth mapping uncertainties), 0.1 Mha is under intensive or extensive (meadow) grasslands (although this is likely to be an overestimate due to mapping uncertainties or deintensification of grazing practices over the last few decades), at least 48,000 ha rewetted and 47,000 ha under peat extraction (Table 1). However, significant uncertainties exist with regard to the spatial mapping of peat per se, and subsequent classification into land use categories due to broad land cover classifications (Evans et al. 2017).

The majority of Scottish peatlands are classified as blanket bogs, a habitat restricted to oceanic climates and high latitudes where cool conditions are found year-round. Scottish peatlands were initially valued as a source of fuel, as evident from 17th-century accounts (Smout 2005). But at the turn of the century, drainage and burning practices to improve grazing became widespread. Early reference to burning of moorland for grazing (‘muirburn’) occurs from 1400, with burning practices continuing to the present (Dodgshon and Olsson 2006). From the late 1780s to the mid-1810s, a radical change in land use took place across Scotland, especially within the Highlands and Islands region, following a shift to a sheep-based economy in the UK that contributed to the Highland Clearances (i.e., large-scale evictions of tenants in the Highlands and Islands areas between 1750 and 1860). The practice of cutting open hill drains on blanket bog to improve productivity and access for grazing livestock has contributed to the large area of modified bog in Scotland (Lilly et al. 2012) (Table 1). A recent study of peatland drainage suggested that over 40,000 km of open hill drains may still exist in the country (MacFarlane et al. 2024).

In coastal areas where people were relocated following the Highland Clearances, peat cutting by hand for domestic fuel was more prevalent (Tindley and Haynes 2014), and domestic peat cutting continues today, albeit in low volumes (Fig. 2b). In addition, peat extraction for distillery use, or to create more arable land was also a common practice around lowland raised bogs. Many of these former peat margins were used to grow local grain crops and have now become grazing lands that are fertilised and sown out with grass species. More recently, land use on some of these former peatland margins has de-intensified.

From the 1940s to the 1980s, large-scale afforestation on deep peat took place in Scotland, primarily driven by tax incentives. This led to the planting of non-native conifers over approximately 20% of Scottish peatlands, including 67,000 ha in the Flow Country of Caithness and Sutherland, the largest expanse of blanket bog in Europe (Stroud et al. 2015). Policy changes in 2015 prohibited afforestation of peat soils deeper than 50 cm and limited reforestation to areas with high yield potential (Forestry Commission Scotland 2015). More recently, the issue of tree-seed rain and non-native conifer regeneration on adjacent peatlands has emerged as another effect of these historical decisions. In Scotland, it is estimated that 267,000 ha of open peatlands are at high risk of encroachment (within 200 m of the forest edge), with a further 579,000 ha at risk within 1 km (The Royal Society of Edinburgh 2024).

## Towards Sustainable Peatland Management, Protection and Restoration

By the 2010s, recognition by researchers, governmental bodies and citizens of the harmful impacts of intensive land-use led to a need for protocols for wiser and sustainable use of peatlands that would enable their protection (Joosten and Clarke 2002). The wide range of ecosystem services that peatlands produce, as well as increasing demand for new conservation areas and improved quality of peatland habitats, was acknowledged both by the wider public and authorities (Rawlins and Morris 2010, Tolvanen et al. 2013, Andersen et al. 2017). For this purpose, Finland, Ireland and Scotland compiled the first national peatland strategies globally (Nordbeck and Hogl 2024). These strategies (MMM 2011, Finnish Government 2012, NPWS 2015, Scottish Natural Heritage 2015) were non-binding documents that guided policy development and have raised awareness among stakeholders (e.g., governmental bodies, peat and forest industry, landowners, and the wider public), although their impact on policies and action has been variable (Nordbeck and Hogl 2024).

More recently, peatland management has been increasingly shaped by national and international policies aimed at addressing the twin crises of climate change and biodiversity loss (Fig. 4). Global biodiversity targets, such as the Aichi Targets of the Convention on Biological Diversity (CBD 2011) and the EU Biodiversity Strategy to 2020 and 2030 (EC 2011, 2020) have driven efforts to halt biodiversity loss and restore degraded ecosystems, with a focus on carbon capture and climate resilience. As part of this objective, the EU Nature Restoration Law (EU 2024) was passed in 2024, albeit with substantially reduced provisions following lobbying efforts, for example, by national farm organisations. In addition, the EU Carbon Removals and Carbon Farming (CRCF) Regulation provides a framework to certify voluntary carbon removals and peatland restoration efforts. Scotland has committed to aligning its environmental standards with the EU. To align with the Nature Restoration Law, a biodiversity strategy with a goal to halt biodiversity loss by 2030 was published in 2024 (Scottish Government 2024b), although the legislation to enact it is still at the early stages in the form of a draft Natural Environment Bill (Scottish Parliament 2025).

Despite these efforts, the conservation status and value of peatland biodiversity are considered poor in all three countries (Table 2), and therefore protection (in terms of designation under nature conservation regulations) alone is not considered adequate (UNEP 2022). In addition, it is recognised that a uniform strategy for conservation and restoration, either across or within countries, is not appropriate. As an example, in Finland, the majority of peatlands considered to be in good ecological condition are located in the northern part of the country, leading to a spatial bias in the needs for conservation versus restoration efforts. For these reasons, peatland restoration has become an elemental part of sustainable management measures.

**Table 2:** Areas (ha) of protected, restored and rewetted peatlands in Finland, Ireland and Scotland in 2023

	Finland (ha)	Ireland (ha)	Scotland (ha)
**Protected peatlands**	**1,280,000**^**a**^	**217,063**^**e**^	**490,216**^**j**^
		**182,660**^**f**^	
Of which state-owned	114,000^a^	41,339	
Of which are raised bogs		17,995	
Of which are blanket bogs		164,665	
**Restored peatlands**	**58,812**	**40,705**	**>43,000**^**k**^
State-owned conservation areas	42,175^b^		
Privately owned conservation areas	1804^b^		
State-owned production forests	10,933^b^		
Private restoration	~3900^c^		
Restored (12 Raised bog SAC only, both public and privately owned)		2649^g^	
Coillte		3311^h^	
Bord na Móna rewetted (as of 2024)		34,745^i^	
*-including restored*		*8100*	
EU LIFE funded			30,000
Peatland ACTION funded			43,000
Peatland CODE			26,000
**Rewetted peatlands/wetlands**	**~3000**	**Unknown**	**Unknown**
Industrial cutaway areas to wetlands	~3000^d^		
Bord na Móna rewetted industrial cutaway areas, plans for 2020–2028 (PCAS)		33,000	

^a^Data from Kaakinen et al. (2018) include ~50,000 ha or 5% drained peatlands

^b^Restored by Metsähallitus (personal communication)

^c^Data obtained from the websites of Hiilipörssi, Snowchange Cooperative and Tornator Oy

^d^Calculated from the Geological Survey of Finland’s peatland site type data: https://hakku.gtk.fi/en/locations/search?location_id=229

^e^National Peatland Strategy (NPWS, 2015) includes SAC (Habitats Directive) and NHA (Natural Heritage Area) under the national Wildlife Acts and ^f^ only contains bogs, not fens

^f^This is less than recorded in 2015 in the National Peatlands Strategy, which was 21,519, as areas have been de-designated https://www.npws.ie/sites/default/files/files/FOR%20UPLOAD%20Plan(WEB_English)_05_02_18%20(1).pdf

^g^Data from https://www.raisedbogs.ie/

^h^EPA (2024) National Inventory Report 2023. Greenhouse gas emissions 1990–2020 Reported to the United Nations Framework Convention on Climate Change. Environmental Protection Agency, Johnstown Castle, Co. Wexford, Ireland, p. 400

^i^Data obtained from Mark McCorry, Bord na Móna, personal communication

^j^Value (from Brown et al. 2023, Evans et al. 2017) contains designated sites with peatlands but also contains habitat mosaics on peat soils that are shallower than the national depth limit of 50 cm and could thus be an overestimate

^k^Value is not certain, since there can be overlaps in the areas counted to EU LIFE, Peatland ACTION and Peatland CODE areas: the highest value of the three is used here

Protected peatlands include national programmes and Natura 2000 areas; restored and rewetted peatlands vary by area type, responsible organisations, and delineation methods

Typically, the term restoration was used as an analogue to ecological restoration, which is defined as an activity with the goal of achieving substantial ecosystem recovery relative to an appropriate reference model, such as native ecosystems (Gann et al. 2019). However, other types of rehabilitation actions that aim to reinstate ecosystem functioning for the provision of specific ecosystem services (Gann et al. 2019), such as paludiculture, are also included in current policies. Paludiculture can be defined as agricultural and forestry systems on peat soils, which aim to conserve the carbon stock of the peat and minimise GHG emissions from the peat soil (Joosten 2024), but it is unclear how effective these systems are in climate protection. Since the mid-1990s (Fig. 3), the availability of EU LIFE funding has been instrumental in the promotion of peatland restoration, whereas more recent approaches to fund conservation and restoration include blended finance models that bring together public finances, as well as voluntary carbon markets and emerging biodiversity credit schemes (Bonn et al. 2014, Moxey et al. 2021).

### Finland

In Finland, a total of 1.3 Mha (~14%) peatlands are within protected areas, although 50,000 ha were drained prior to protection, resulting in continued degradation (Kaakinen et al. 2018). The first targeted National Mire Conservation Programme was implemented in 1981 (Fig. 3) and covered 600,000 ha (Kaakinen and Salminen 2006). Peatlands are also protected within national parks and other conservation areas. Since the establishment of the EU Natura 2000 network in 1998, an additional 35,000 ha of peatlands have been designated for conservation. Despite the relatively high proportion of protected peatlands, 54% of peatland habitat types are classified as *endangered*, and 20% are considered *near-threatened* (Kontula and Raunio 2019), with nutrient-rich habitats in the southern regions most affected. Notably, the geographical distribution of protected and undrained peatlands is strongly biased towards the northern part of the country.

Created in the early 2010s, the national peatland strategy of Finland was the first (globally) to adopt an ecosystem services approach, combining environmental, social and economic targets for responsible peatland use (MMM 2011, Finnish Government 2012). The strategy focused on guiding new land use changes (particularly peat extraction) into peatlands that had already lost their natural state, i.e., areas impacted by drainage. In 2015, changes in the government (after the departure of the pro-nature Green party) led to a controversial development in legislation (Act 513/2015), where the planned increase in the tax on peat energy use was revoked, and further decreases in taxes were to follow. In addition, the preparation of a Supplemental Mire Conservation Programme (Alanen and Aapala 2015), which would have designated 117,000 ha of new peatland conservation areas, was halted (Albrecht 2018). Despite these signs of weaker peatland protection, the national restoration target was set at 15% in 2015 (Kotiaho et al. 2015, 2016), which aligned with the EU Biodiversity Strategy to 2030 (EC 2020).

Restoration efforts had already started in the 1970s (Fig. 3) in protected areas (Aapala et al. 2013), with a significant increase in projects following the availability of EU LIFE funding in the mid-1990s. Restoration became an established element of the management of protected areas under the nationwide targets set by the Finnish Board on Ecological Restoration (established in 2004) and by the launch of the Forest Biodiversity Programme for southern Finland METSO in 2008 (Finnish Government 2008).

**Fig. 5:**
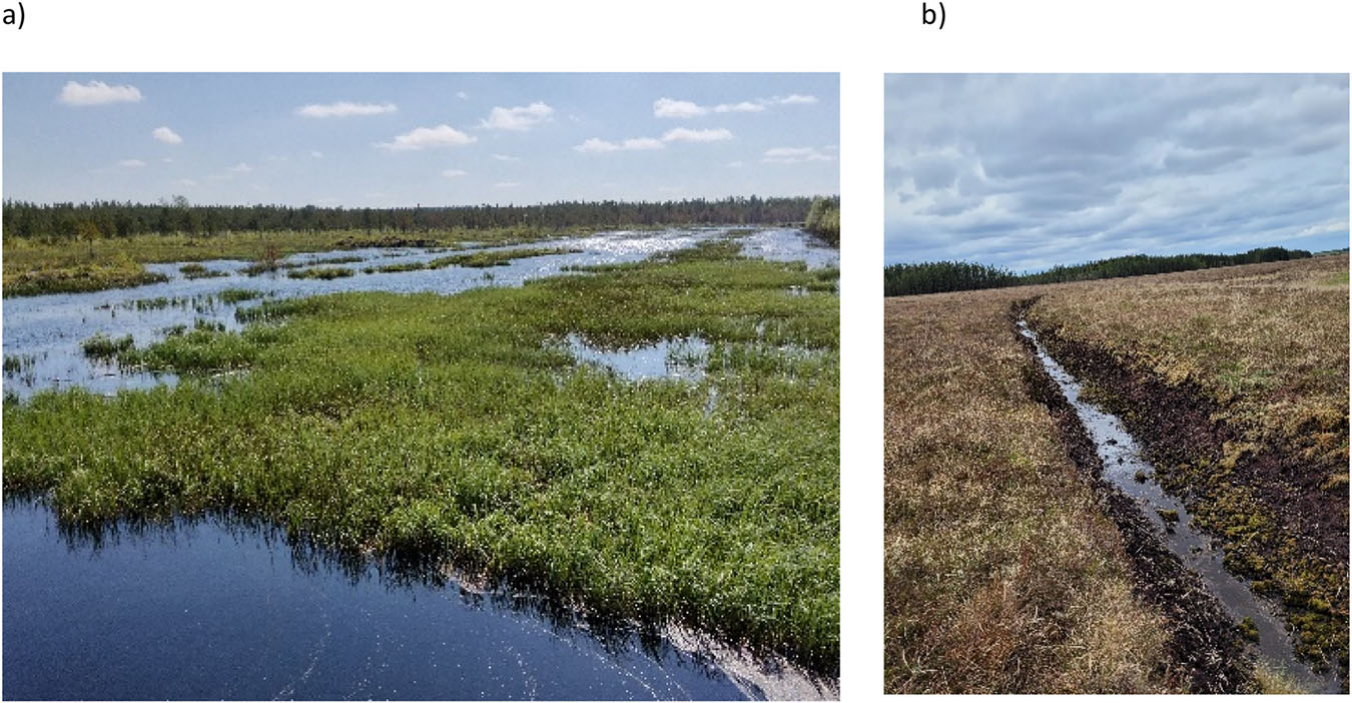
**a** Rewetted industrial cutaway peatland in Finland (photo: Anna Laine-Petäjäkangas), **b** active drainage of blanket bogs for agricultural use in Ireland (photo: David Wilson)

Peatland restoration in Finland has mainly focused on forestry-drained peatlands. The emphasis has been to restore conditions as close to natural as possible, thereby enhancing the quality of biotopes and habitats, and halting biodiversity loss (Aapala et al. 2013). A monitoring network in the areas restored by Metsähallitus, the managing body for state-owned lands, was established early on to evaluate the success (or otherwise) of the measures (Elo et al. 2024). In addition, small-scale rewetting of industrial cutaway peatlands (~3000 ha) has created open wetland habitats that are especially suitable for waterbirds (Fig. 5). Depending on their management (some are intentionally kept open to support waterbirds), these sites may offer a starting point for hydroseral succession towards a peatland ecosystem. Most restoration projects have been carried out by Metsähallitus, but in recent years, restoration has also been carried out by private entities with support from the State via the Helmi habitats programme (2021–2030, Gummerus-Rautiainen et al. 2021). Especially in private lands, fragmented landownership has limited the size of the restoration areas, and higher impact catchment-scale restoration with multiple benefits related to hydrological integrity, biodiversity and carbon conservation is hard to achieve. New, binding, area-based restoration targets were set by the EU Nature Restoration Law.

Peatland land use in the country is currently guided by several recent national policies. The *Carbon Neutral Finland by 2035* policy, which aims to reduce peat energy use by 50% by 2030 (Finnish Government 2019, TEM 2022), was reached ahead of schedule in 2021. This rapid change came as a shock to the peat industry, and financial compensation for the loss of business was sought from the EU Just Transition Fund (Korhonen et al. 2021). In addition, the Finnish government launched a Climate Plan for the Land Use Sector in 2022 (MMM 2023) with a specific target of reducing GHG emissions by at least 3 Mt carbon dioxide equivalents (CO_2_-eq) per year by 2035, by additional measures in the land use sector, including a rather strong emphasis on peatlands. This would include, for example, raising water levels in peat fields, creation of wetlands in cut-over peatlands, continuous cover forestry and promotion of ash fertilisation in peatland forests.

### Ireland

Less than 15% of the original raised bogs and 23% of the original blanket bogs in Ireland have been protected under the Habitats or Birds Directives. While designation started in the late 1980s (for birds) and the late 1990s (for habitats), these directives were officially incorporated into Irish law by the European Communities (Birds and Natural Habitats) Regulations of 2011, with subsequent amendments.

In Ireland, the establishment of the Peatlands Council in 2011 and the development of the first National Peatlands Strategy (NPWS 2015) with the aim of balancing traditional turf cutting rights with conservation efforts (Fig. 4). The strategy raised awareness around peatland use, balancing the needs and interests of the entire peatland stakeholder community^3^ (Flood et al. 2025), while committing governmental institutions to build crucial knowledge capacity (funding research programmes and communities’ schemes; Renou-Wilson et al. 2022, Wilson et al. 2022). Through the work of the Peatlands Council and the restoration and rehabilitation of Bord na Móna bogs, as well as EU LIFE and Just Transition funding, the Irish State aspires to embed the sustainability principles into future management, policy and planning of its largest natural resource, peatlands.

Peatland restoration in the country is still in its early stages, and fragmented landownership can hold back the formation of larger-scale initiatives. Restoration efforts have focused on: (1) restoration of designated raised bogs SACs (started with the Irish Peatland Conservation Council and continued with the Living Bog project under the EU LIFE programme 2016–2022) with 12 SAC sites (2600 ha) being restored (The Living Bog, 2016); (2) rewetting of drained bogs that had not been extracted by Bord na Móna, and cutaway peatlands where natural regeneration of peat-forming vegetation had occurred; (3) blocking of drainage and removal of failed conifer crops from afforested sites owned by Coillte under the EU LIFE programme; (4) restoration efforts led by local communities and NGOs (e.g., Irish Peatland Conservation Council) alongside other conservation programmes that aimed to protect species, such as the Hen Harrier (http://www.henharrierproject.ie/) or the Freshwater Pearl Mussel (https://www.pearlmusselproject.ie/). These programmes are supported by the Result-Based Agri-Environmental Payment Scheme (RBAPS), which has been expanded under EU LIFE (https://www.wildatlanticnature.ie/) and European Innovation Partnership projects (https://www.farmpeat.ie/). In short, the overall aim of RBAPS is to financially reward farmers for achieving and maintaining verifiably high-quality ecological conditions that ensure C storage, biodiversity and clean water, rather than just paying for prescribed actions.

Despite the past efforts, in the most recent five-year assessment, all Irish peatland habitats were classified as having ‘*Unfavourable-Bad’* conservation status (NPWS 2019). Alarmingly, this declining trend has persisted since the onset of reporting (Fig. 5), with ongoing domestic turf cutting on protected SAC sites contributing to further degradation.

Since 2023, Bord na Móna has ceased all peat extraction for energy purposes. The company received substantial State funds (€108 million) to rewet and rehabilitate around 33,000 ha of its land (Irish Government 2020). Unlike previous efforts that focused on stabilising peat and mitigating silt runoff, this enhanced rehabilitation involves rewetting through bunding and drain blocking, as well as additional restoration techniques, such as Sphagnum reintroduction and paludiculture demonstration plots. In addition, local community engagement has grown with organisations, such as the Community Wetlands Forum (established in 2013), which advocate for sustainable peatland management through active community involvement (Flood et al. 2022).

The Nature Restoration Law (NRL) and CAP reform are expected to drive significant rewetting of peat soils, including areas previously used for peat extraction or abandoned. The focus will likely be on those sites where landowners are most open and willing to participate in schemes such as the Result-Based Agri-Environmental Payment Scheme (RBAPS). Ireland has improved its GHG reporting for UNFCCC through Tier 2 estimates for land-use categories, including domestic peat extraction (EPA 2024), and a new near-natural wetlands category based on Irish GHG studies (Aitova et al. 2023).

### Scotland

In the late 1990s, the EU Habitats Directive provided a mechanism to protect and designate important peatland areas in Scotland (Fig. 3). In Scotland, the fact that about half of the land is owned by fewer than 500 individuals or companies has facilitated some larger-scale conservation and restoration initiatives over the last few decades. EU LIFE funding enabled large-scale restoration efforts for the first time alongside newly established SAC and Special Protection Areas (SPA). One of the earliest initiatives was a forest-to-bog management project at the RSPB Forsinard Flows National Nature Reserve in 1998–99, driven by concerns over the negative impacts of afforestation on biodiversity in the Flow Country, especially on breeding waders (Wilson et al. 2014). This was followed by more initiatives that contributed to a 2014 policy change that prohibited afforestation on sites where peat depth is >50 cm and mandated the removal of forestry adjacent to designated peatlands. This has resulted in the removal of non-native conifers from large areas of peatland across the country. By 2015, more than 30,000 ha of peatlands had undergone restoration and management interventions funded by the EU LIFE projects (Andersen et al. 2017).

This policy change was seen in Scotland’s National Peatland Plan from 2015 (Fig. 4) that aimed to “protect, manage and restore peatlands” (Scottish Natural Heritage 2015) and set ambitious targets for restoration by 2020, 2030 and 2050 (Scottish Government 2013) and was seen as a national strategy for the conservation and enhancement of biodiversity in Scotland. In recognition of the role of peatland restoration in reducing GHG emissions, Scotland launched the Peatland ACTION Programme in 2012, following a 2011 review of UK peatland conditions by the IUCN Peatland Programme (Bain et al. 2011). The Peatland ACTION programme, along with the 2015 National Peatland Plan, outlined a long-term vision for healthy and resilient peatlands across Scotland. At the same time, the Peatland Code, the first mechanism to attract private investment for GHG emissions reductions through restoration, was introduced across the UK.

By 2020, progress was made in funding restoration, carbon protection, increasing peat-free compost use, and improving conditions in protected peatland areas (Scottish Government 2023c, 2024c, NatureScot 2025b). Initially, the scale and pace of implementation were slower than expected, in part because of a lack of uptake of public funding schemes from landowners. As uptake increased, the lack of skilled contractors to meet the demand became a new bottleneck. In 2020, £250 million was pledged by the Scottish Government to support restoration and management efforts, with a challenging goal to restore 250,000 ha of peatlands by 2030 (Scottish Government 2020). By 2023, over 200 projects had been initiated under the Peatland Code, driven by a combination of private and public funding (primarily from Peatland ACTION). In 2023, the areas restored through the first EU LIFE projects in the Flow Country were formally integrated into the Peatlands of Caithness and Sutherland SAC. This was a key milestone made possible by the emerging body of evidence that had demonstrated the trajectory of these areas towards functioning blanket bog systems, including in terms of biodiversity (Pravia et al. 2020, Hughes et al. 2025), hydrology (Gaffney et al. 2018) and carbon sequestration (Hermans et al. 2019).

To date, there are no reliable statistics available on the area that has benefited from the improved management or restoration (Table 2), as the current reporting mechanism only reports on the actions (i.e., the areas that have received management interventions) rather than outcomes. While there is evidence from local-scale restoration projects that rewetting efforts have improved hydrological dynamics, vegetation composition, and wider habitat quality or GHG emissions (e.g., Lees et al. 2019, Haycock and Cashon 2022, Ball et al. 2023, Burdun et al. 2025, Hughes et al. 2025, Large et al. 2025), a standardised monitoring of ecosystem functions has not been deployed consistently across all the sites and would include pre-restoration baseline and reference controls. A monitoring strategy has been developed more recently (Artz et al. 2023, NatureScot 2025a), and several initiatives to provide large-scale assessments of changes in conditions following restoration have been funded (e.g., Artz et al. 2019, Large et al. 2025).

A key achievement in Scottish peatland conservation occurred in July 2024, when the Flow Country became the first and only peatland globally to be recognised as a UNESCO World Heritage Site. This sets a global precedent for peatlands, and will need to be rigorously monitored (species, key features such as pool systems, ecosystem functions, threats, management) to comply with IUCN requirements.

Currently, there are a number of environmental policies and legislation in existence in relation to peatlands. A new piece of legislation was passed in 2024 in relation to the management of muirburn (controlled burning of moorland vegetation, which often includes areas of upland, modified peatland based on a depth-based definition of peat, i.e., >40 cm in Scotland) and wildlife management, which includes a new licensing scheme for muirburn application to be introduced in autumn 2025 (Scottish Parliament 2024). There is also an intention to ban the sale of peat in Scotland, initially for home compost use (Scottish Government 2023c). In the voluntary markets, there are plans to include biodiversity credits in the UK Peatland Code in 2025, to support restoration through existing and new public-private partnerships (IUCN 2024a). Peatland management is also referred to in planning policy, where, for example, the National Planning Framework 4 (Scottish Government 2023b) includes a range of policies relating to peatlands, their protection and restoration. However, the protection, restoration and mitigation hierarchy on peatlands is not weighted equally across all developments and can be overruled in the case of nationally critical energy infrastructure project developments.

## Discussion

The historical and ongoing exploitation of peatlands in Finland, Ireland and Scotland poses threats and challenges to biodiversity conservation, climate regulation and water quality. Peatland use and exploitation have centuries-long roots in all three countries and are ingrained in 20th-century national narratives of transforming unproductive “backcountry” into productive and prosperous landscapes. Land-use pressure causes fragmentation of naturally functioning peatlands and compromises their inherent ecological value and functions. The decline in the ecosystem services provided by peatlands has been well documented and ranges from accelerated ombrotrophication and shrubification in the margins of sites (Tahvanainen 2011, Kolari et al. 2022) to lowered water tables with an increasing risk of switching the undrained peatlands from net carbon sinks to net sources during drought periods (Helbig et al. 2020). Moreover, the increased drought frequency (driven by climate change) has highlighted the importance of naturally functioning peatlands for water security in both Scotland and Ireland (Sabokrouhiyeh et al. 2025). Drier peatlands are also more susceptible to wildfires (Wilkinson et al. 2023), which can cause substantial carbon emissions, air and water pollution, biodiversity losses, and have profound impacts on local communities.

The significantly decreased and fragmented area of undrained and actively carbon accumulating peatlands requires urgent action, and mere protection of these remnant peatlands is insufficient. The potential for peatland restoration as a nature-based solution to mitigate climate change has long been proposed (Parish et al. 2008, Joosten et al. 2012) and in recent years has been validated by empirical (Renou-Wilson et al. 2019, Günther et al. 2020, Wilson et al. 2022, Bockermann et al. 2024), modelling (Humpenöder et al. 2020) and meta-analysis studies (Darusman et al. 2023). In addition to restoration, other rehabilitation practices have been promoted to decrease the pressure on peatlands. These actions typically rely on rewetting or raising the water table to a depth that decreases peat decomposition, e.g., paludiculture or peatland farming and forestry with higher water tables (Chen et al. 2023). The conversion of arable peatland to grassland or the adoption of continuous cover forestry on commercial peatland forests could also reduce environmental impacts, as both rely on more or less continuous vegetation cover, and can be practised with higher water levels (wetter conditions) (Nieminen et al. 2018, Lehtonen et al. 2023). However, we are now in a time of transition due to changes in regulations and markets. Indeed, sustainable peatland management requires a regime shift towards improved hydrological and ecological conditions. Despite the steps taken during the 2010s towards more sustainable peatland management in the three countries, evidence of improvements in the ecological state of peatlands is largely missing. Here, we discuss the steps needed and barriers to overcome to achieve sustainable peatland management and suggest potential solutions.

### Implementation of an Integrated, Multi-stakeholder, Landscape-scale Strategy

Effective sustainable peatland management requires integrated landscape/catchment-scale planning to ensure adequate water availability, habitat connectivity, and impactful outcomes for water quality and water flow regulation (Temmink et al. 2023). The three countries have notable differences in their landownership structure related to peatlands. In Finland and Ireland, small and scattered landholdings complicate the planning of hydrologically integrated management practices, such as restoration and paludiculture. In a landscape with fragmented landownership, the formation of sufficiently large entities is difficult. Negotiations are needed with a multitude of parties, and typically, one landowner can block the entire process. In Scotland, where landownership is concentrated in the hands of a small number, large-scale restoration efforts have been possible but have also raised concerns about equitable outcomes under the Just Transition policy (NatureScot 2025a). Regardless of the land tenure structure, collaboration between the landowners, policy makers, practitioners, researchers, planners and users of the landscapes is essential to develop socially and ecologically sustainable land-use plans. Pilots of such “Regional Land Use Partnerships”, designed to create better connected landscapes that support habitats (not limited to peatlands), improve water quality and reduce flood risk amongst other benefits, have been implemented in Scotland in five regional schemes with promising results (Scottish Government 2025a, Stevens 2025).

### Monitoring of Restoration Outcomes Is Needed

To ensure the ecological effectiveness of the actions, evaluation of the impact of restoration measures is crucial (Andersen et al. 2017). However, monitoring approaches vary between countries, restoration programmes and individual projects, and there is a lack of consensus on restoration success indicators, and often a lack of suitable reference ecosystems or baseline data on pre-restoration dynamics (Renou-Wilson et al. 2025). In Scotland, restoration has been largely driven by landowners’ demands rather than by a coordinated strategy, and although monitoring programmes are incorporated at some sites, the data may not be sufficiently robust to untangle the compounding effects of landscape, climate, restoration techniques or other variables on the outcomes (Artz et al. 2020). However, a monitoring framework, as well as funding, has also been recently deployed across networks of sites and for site-specific monitoring of processes (e.g., hydrology, GHG emissions) and/or species, with data integrated in a national, openly available data repository (Artz et al. 2023, NatureScot 2025a, Large et al. 2025). Unlike in Scotland, Finland’s restoration efforts, thus far, have mainly focused on state-owned land managed by Metsähallitus, and are supported by a long-term monitoring network that provides information to improve practices and cost-effectiveness (Ikkala and Similä 2024). However, methods for monitoring outside state-run projects and funding schemes are largely lacking. Upscaling restoration, which would involve a multitude of financing sources and actors, would benefit from coherent, long-term monitoring frameworks coordinated by, for example, governmental (research) organisations (Flood et al. 2025). This will become essential when EU countries begin to report restored areas needed to achieve the goals set by the EU Nature Restoration Law (EU 2024).

### Integrated Policies to Promote Sustainable Peatland Use

Finland, Ireland and Scotland were the first countries to create national peatland strategies with an emphasis on the balance between peatland use and protection. However, the pace of climate change and the biodiversity crisis has rendered these strategies outdated. For better policy steering, future peatland and catchment management strategies must integrate more nature-based solutions to keep pace with these accelerating changes (Nordbeck and Hogl 2024).

One clear barrier to sustainable peatland management in all three countries is the lack of policy coherence at national and EU levels. Climate change mitigation efforts are often undermined by subsidies that promote peatland drainage (Fig. 5) or tree planting. In Finland, the Water Act is skewed towards drainage, so that the drainage of a neighbour’s land is, by default, allowed, while rewetting is prohibited. In Scotland, the issues generated by non-native conifers spreading outside of plantations and onto adjacent areas of peatland currently have no clear long-term solution or financial mechanism to support their removal. In the agricultural sector, most plant species that are suitable for paludiculture do not qualify for Common Agricultural Policy (CAP) payments, thereby limiting financial support for more climate-friendly practices (Regina et al. 2016, Wichmann and Nordt 2024).

Better sectoral coherence and policy integration with regard to climate, biodiversity, energy and land use sectors are necessary to transition towards sustainable peatland use (Chen et al. 2023). This would mean review and reformation of existing incentives and subsidised land uses to support and reward carbon sequestration and biodiversity conservation initiatives instead of continued or new peatland drainage for agriculture, afforestation, horticultural peat and peat energy production. In areas with fragmented land ownership, land consolidation protocols developed for agriculture and forestry (e.g., landowners in Finland can apply to the National Land Survey for a land consolidation arrangement that combines small and often narrow parcels into more manageable entities) could be used to create suitable target areas for peatland restoration. The new EU Nature Restoration Law (EU 2024), with national restoration plans with detailed targets under development and reserved funding, also presents an opportunity for large-scale peatland restoration. In Scotland, large-scale peatland restoration is already incentivised through existing programmes, as larger areas are more likely to yield economies of scale at the point of intervention and are more likely to be profitable in terms of carbon credits.

### Decrease the Trade-off Between Green Energy Transition and Sustainable Peatland Use

The green transition, driven by ambitious national and EU-level targets, offers an opportunity to change peatland use for the better. Under the EU RED III Directive (EU 2023), Finland and Ireland must ensure that renewable energy accounts for at least 42.5% of final energy consumption by 2030. Finland is already ahead of the target at 43.5%, largely due to a high share of wood-based fuels, while wind and solar together contribute only about 6% (Statistics Finland 2025). In contrast, Ireland remains far behind with renewables at 5% of the total energy use (CSO 2025). Scotland has set a target for 50% of the total energy use from renewables by 2030 (Scottish Government 2020), compared to the current level of 30% (Scottish Government 2025b). If unregulated, the green transition can also mean the adoption of new harmful peatland uses with habitual continuation of old drainage practices when new areas for wind and solar farms are sought. The economic profit for a private landowner to sell or rent land for energy companies easily outcompetes restoration schemes. In Scotland and Ireland, blanket peatlands have been used as sites for wind farms for decades (Renou-Wilson and Farrell 2009, Chico et al. 2023). The often-remote blanket peatlands have manifold problems, such as landslide vulnerability, fragmentation of hydrological units and peat subsidence that cause ecosystem degradation and carbon losses, thus their suitability as a location for wind farms has been questioned (Smith et al. 2014 and 2021). In Scotland, as some of the early wind farms on peat are nearing end-of-life, there are now questions around the barriers and opportunities offered by repowering, i.e., the re-use of existing developments instead of decommissioning (Waldron et al. 2018, Philpott and Windemer 2022). These challenges are recognised in Scotland’s current National Planning Framework, which emphasises the need to balance essential infrastructure requirements with peatland restoration and emissions mitigation targets at the local level (Scottish Government 2023b). The abandoned peat cutaways in Ireland and Finland are seen as good locations for renewable energy by companies, governments and landowners, since in their current state they are considered unproductive industrial land (NPWS 2015, Finnish Government 2023). Whether biodiversity and soil carbon stocks can be promoted hand-in-hand with renewable energy at the same sites remains to be seen; thus, solar power plants with wetland uses, such as *Sphagnum* moss cultivation, should not be mutually exclusive (Ikkala et al. 2025). However, comprehensive environmental and socio-economic impact assessments must precede interventions, with strict enforcement of minimal ongoing peatland drainage and mandatory rewetting of degraded areas. The mitigation hierarchy involving avoidance, minimisation, and compensation phases should be used to reduce the negative effects of wind and solar power on biodiversity (Tolvanen et al. 2023).

### Engaging Local Communities in Restoration

The three countries offer rather different approaches to governance of restoration. In Finland, the approach has been state-led and top-down, but the view is changing, and state-run funding programmes, such as METSO and HELMI, have also promoted restoration on privately owned land. However, the launching of new finance mechanisms seems slow, and while HELMI programme funding has been used quite efficiently to restore peatlands within protected areas, the use in privately owned land during the first four years of funding has been minimal (YM 2025). In Ireland, the approach has been partly top-down (Habitats Directive) and bottom-up, with all the Irish stakeholders, including the governmental agencies, NGOs, the agricultural (including forest) sector and an increasing number of local communities recognising the environmental costs of drained peatlands and the benefits of rewetting (Gibson-Graham and Dombroski 2020, Flood 2023). It is understood that the successful transition to these new sustainable systems will require the co-creation of context-appropriate solutions that are based on socio-ecological investment and resourcing that cover both scientific monitoring and collaborative networks between all the stakeholders with meaningful community engagement (Flood 2023).

### Promotion of Voluntary Markets

The voluntary carbon market may provide an alternative funding source for peatland restoration. In recent years, the utilisation of rewetted peatlands in voluntary carbon offset projects has been made possible by the development of methodologies for the monitoring, validation and verification of GHG emissions savings (e.g., Evans et al. 2022, Emmer & Couwenberg 2017, IUCN 2024b). The UK, with their domestic voluntary carbon market standard, The Peatland Code, which started in 2015, has the longest experience in peatland carbon trading, with outcomes yet to be validated. The technical complexity of the Peatland Code process, combined with the wider global issues around carbon trading, has created hesitancy amongst landowners and investors. In Scotland, it has also brought up issues around wealth distribution and societal inequalities, for which innovative solutions need to be developed. In Finland, the voluntary carbon markets play a minor role in the Finnish land use sector. The greatest areal potential is in forestry-drained peatlands, yet the emission reductions cannot be guaranteed with restoration, especially on nutrient-poor site types (Laine et al. 2024). To date, the peatland carbon market in Ireland remains unregulated, and a “Wild West” trading environment has developed. However, the establishment of a Peatland Carbon Code, underpinned by a Peatland Finance Ireland initiative, will offer bundled carbon-biodiversity-water quality packages to potential investors, potentially by 2026 (Cox 2023). In Scotland, there are plans to include biodiversity credits in the UK Peatland Code in 2025, to support restoration through public-private partnerships (IUCN 2024a). Yet, the voluntary biodiversity markets are still forming, and their potential as a funding source for peatland remediation is unknown.

## Conclusion

To achieve sustainable peatland conservation and management, coordinated efforts are needed to address the ecological, political and socio-economic barriers identified in Finland, Ireland and Scotland. Comprehensive restoration and rehabilitation strategies with long-term funding, supported by updated national policies and international frameworks, such as the EU Nature Restoration Law, will be critical in preserving these vital ecosystems. Peatland management must evolve to meet the demands of climate resilience and biodiversity conservation, and alternative land-use options, such as paludiculture and voluntary carbon and biodiversity markets, can play a key role in ensuring their long-term sustainability. Governmental subsidies and regulatory frameworks have historically contributed to the degradation of peatlands in all three countries. While corrective measures have been introduced, their approaches vary. In Scotland, the development of private financing mechanisms for restoration represents a promising avenue for scaling efforts. In Finland, robust governmental systems for monitoring restoration areas and success could be extended to private landowners and adapted for use in other countries. In Ireland, by contrast, emphasis has been placed on engaging local communities and promoting co-creation in restoration planning and implementation. Integrating these strategies could accelerate learning and support progress towards achieving a favourable conservation status, as outlined in instruments such as the Nature Restoration Law. 4

## Data Availability

No datasets were generated or analysed during the current study.

## Footnotes

1 Scotland was chosen for this comparison (Brown et al. 2023) as it has an extensive cover of peatlands (~2 million ha, which equates to 66% of UK peatlands). While net GHG emissions from peatlands are reported to the UNFCCC for the UK as a whole, the Scottish Parliament, as a devolved administration from the UK, has the power to make laws related to environmental management and protection (including peatlands). Therefore, the Scottish Government is responsible for the formulation and implementation of policies in these areas.

2 Definition of the sustainable management of peatlands follows Flood et al. (2022) (“Sustainable peatland management is defined as societal responsibility to optimise the social, cultural, environmental, and economic contributions that peatlands make to the health and well-being of current and future generations”) and UNEP (2022) (see terms “Sustainability” and “Sustainable use” in the Glossary).

3 Stakeholder communities include local communities, landowners, researchers, governmental and NGO communities; see mapping of stakeholders in Pschenyckyj et al. (2021).

4 Scottish Natural Heritage changed its name to NatureScot in 2020, and documents are currently found on NatureScot websites.
